# iPSC‐Derived Liver Organoids as a Tool to Study Medium Chain Acyl‐CoA Dehydrogenase Deficiency

**DOI:** 10.1002/jimd.70028

**Published:** 2025-04-08

**Authors:** Ligia A. Kiyuna, José M. Horcas‐Nieto, Christoff Odendaal, Miriam Langelaar‐Makkinje, Albert Gerding, Mathilde J. C. Broekhuis, Flavio Bonanini, Madhulika Singh, Dorota Kurek, Amy C. Harms, Thomas Hankemeier, Floris Foijer, Terry G. J. Derks, Barbara M. Bakker

**Affiliations:** ^1^ Laboratory of Pediatrics University Medical Center Groningen, University of Groningen Groningen the Netherlands; ^2^ Department of Laboratory Medicine University Medical Center Groningen, University of Groningen Groningen the Netherlands; ^3^ European Research Institute for the Biology of Ageing University Medical Center Groningen, University of Groningen Groningen the Netherlands; ^4^ Mimetas Leiden the Netherlands; ^5^ Metabolomics and Analytics Centre Leiden Academic Centre for Drug Research, Leiden University Leiden the Netherlands; ^6^ Section of Metabolic Diseases, Beatrix Children's Hospital University Medical Centre Groningen, University of Groningen Groningen the Netherlands

**Keywords:** coenzyme a, iPSC, MCADD, medium‐chain acyl‐carnitines, organoids, peroxisomes

## Abstract

Medium chain acyl‐CoA dehydrogenase deficiency (MCADD) is an inherited metabolic disease, characterized by biallelic variants in the *ACADM* gene. Interestingly, even with the same genotype, patients often present with very heterogeneous symptoms, ranging from fully asymptomatic to life‐threatening hypoketotic hypoglycemia. The mechanisms underlying this heterogeneity remain unclear. Therefore, there is a need for in vitro models of MCADD that recapitulate the clinical phenotype as a tool to study the pathophysiology of the disease. Fibroblasts of control and symptomatic MCADD patients with the c.985A>G (p.K329E) were reprogrammed into induced pluripotent stem cells (iPSCs). iPSCs were then differentiated into hepatic expandable organoids (EHOs), further matured to Mat‐EHOs, and functionally characterized. EHOs and Mat‐EHOs performed typical hepatic metabolic functions, such as albumin and urea production. The organoids metabolized fatty acids, as confirmed by acyl‐carnitine profiling and high‐resolution respirometry. MCAD protein was fully ablated in MCADD organoids, in agreement with the instability of the mutated MCAD protein. MCADD organoids accumulated medium‐chain acyl‐carnitines, with a strongly elevated C8/C10 ratio, characteristic of the biochemical phenotype of the disease. Notably, C2 and C14 acyl‐carnitines were found decreased in MCADD Mat‐EHOs. Finally, MCADD organoids exhibited differential expression of genes involved in ω‐oxidation, mitochondrial β‐oxidation, TCA cycle, and peroxisomal coenzyme A metabolism, particularly upregulation of *NUDT7*. iPSC‐derived organoids of MCADD patients recapitulated the major biochemical phenotype of the disease. Mat‐EHOs expressed relevant pathways involved in putative compensatory mechanisms, notably CoA metabolism and the TCA cycle. The upregulation of *NUDT7* expression may play a role in preventing excessive accumulation of dicarboxylic acids in MCADD. This patient‐specific hepatic organoid system is a promising platform to study the phenotypic heterogeneity between MCADD patients.

## 1 Introduction

Medium‐chain acyl‐CoA dehydrogenase (MCAD) is one of the flavoenzymes that catalyze the first step of mitochondrial β‐oxidation of fatty acids. It oxidizes medium‐chain acyl‐CoA into 2‐enoyl‐CoA. MCAD deficiency (MCADD) is an autosomal recessive disease and the most common fatty acid oxidation disorder (FAOD) with a prevalence of 1/8300 in the Netherlands [1]. Interestingly, people homozygous for the prevalent c.985A>G (p.K329E) *ACADM* variant can present various clinical phenotypes. While some patients remain asymptomatic throughout life, some experience life‐threatening hypoketotic hypoglycemia when exposed to catabolic stress (e.g., fasting and intercurrent illness) [2, 3, 4]. After diagnosis, the most common treatment relies on avoidance of fasting and an emergency regimen [5, 6]. While this variant is estimated to account for more than 90% of pathogenic alleles, the implementation of MCADD in population newborn screening programs has revealed several *ACADM* variants of unknown clinical significance [7]. The clinical risk associated with individual variants and the mechanisms underlying metabolic decompensation remain unclear for individual patients [7, 8]. Patients with residual MCAD activity, equal to or above 10%, have been referred to as mild and often remain asymptomatic [1]. However, within the cohort of c.985A>G (p.K329E) homozygotes, patients may present with a wide range of symptoms and disease severity, suggesting a limited genotype–phenotype correlation. Therefore, it is likely that genetic variation beyond the *ACADM* gene, the environment, and epigenetics play a major role in the development of a metabolic crisis [9].

Preclinical, mechanistic studies of the pathophysiology underlying MCADD have traditionally relied on the use of animal models [10, 11, 12]. However, rodents are equipped with an extra dehydrogenase, long‐chain acyl‐CoA dehydrogenase (LCAD), which shows overlapping substrate specificity with MCAD [13] and may therefore mask the phenotype. Additionally, a full deletion of the functional MCAD enzyme cannot elucidate the effect of individual point mutations in the *ACADM* gene. This makes human and patient‐derived in vitro models an attractive alternative for the study of MCADD. Organoids are 3D multicellular structures that proliferate in vitro while recapitulating several functions of the organ of origin. They can be obtained from primary tissue as well as from pluripotent and adult stem cells [14]. Liver organoids recapitulate several hepatic functions such as albumin production, bile acid production, and CYP3A4 activity [15]. Since they were first described [16], organoids have been used for an increasing number of applications including the study of differentiation, organ development [17], and (metabolic) disease [18], as well as drug screening [19]. For primary tissue, highly invasive liver biopsies are required. In contrast, hepatobiliary organoids can also be derived from induced pluripotent stem cells (iPSCs) [20, 21, 22, 23]. The latter can be obtained from fibroblasts, lymphoblasts, or even urine cells, providing an expandable source of hepatic cells through a minimally invasive procedure [24]. Several protocols have been developed for differentiating iPSCs into hepatobiliary organoids. Although these organoids remain closer to fetal than to adult tissue [25, 26, 27], they represent an important organ‐specific system to understand not only the disease mechanism but also patient‐specific phenotypes and symptomatology.

Patient‐specific iPSC‐derived MCADD hepatobiliary organoids could serve (i) to study the effect of different mutations in the *ACADM* gene itself and (ii) to study compensatory mechanisms that may depend on genetic variation outside the *ACADM* gene. To realize this goal, it is important that organoids recapitulate the major biochemical phenotype. Moreover, pathways that have been hypothesized to play a compensatory role in asymptomatic patients should be active in the organoids. Putative compensatory enzymes and pathways include short‐chain acyl‐CoA dehydrogenase [28], peroxisomal β‐oxidation [29] and coenzyme A metabolism [28]. Whereas the MCAD enzyme is localized in the mitochondria, peroxisomes are single‐membrane organelles equipped with their own β‐oxidation pathway. They are involved in many metabolic processes and are highly abundant in liver cells. They are renowned for their ability to oxidize branched‐chain and very‐long‐chain fatty acids [30], yet they are also capable of oxidizing medium‐chain fatty acids (MCFA) [29, 31] and fatty dicarboxylic acids [32]. To the best of our knowledge, there are currently no experimental studies reporting on the role of peroxisomes in any of the deficiencies of mitochondrial dehydrogenases (SCADD, MCADD and VLCADD).

The goal of this study was to establish and characterize an iPSC‐derived hepatobiliary organoid system for the study of MCADD. iPSCs derived from fibroblasts of symptomatic MCADD patients with the classical c.985A>G (p.K329E) mutation were differentiated into hepatic organoids and compared to organoids from healthy controls. Here we demonstrate that MCADD organoids recapitulate typical diagnostic MCADD metabolites. Moreover, mature organoids upregulate peroxisomal markers, making them a suitable system to study patient‐specific differences in peroxisomal metabolism. Finally, we report a minor regulation of peroxisomal CoA metabolism in organoids derived from symptomatic MCADD patients.

## 2 Results

### 2.1 Generating Induced Pluripotent Stem Cells From Control and MCADD Patient Fibroblasts

Control and MCADD fibroblasts were reprogrammed into iPSCs following a previously published protocol [33]. Immunofluorescence confirmed the expression of NANOG, OCT4, SOX2, SSEA‐4, Tra‐1‐60, and Tra‐1‐81, indicating the pluripotency of the iPSCs (Figure S1).

### 2.2 Generating Expandable Hepatic Organoids (EHOs) From Control and MCADD iPSCs

**FIGURE 1:**
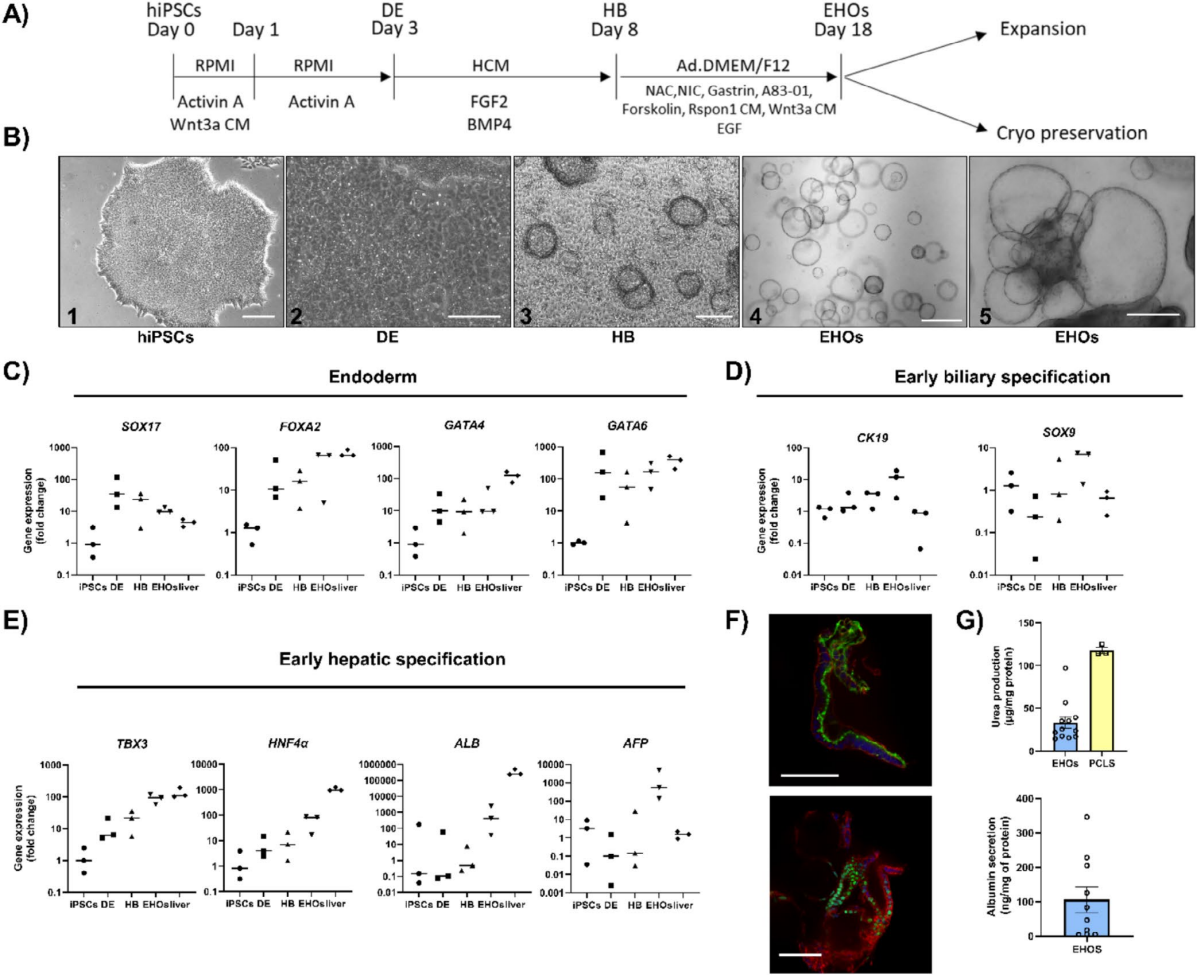
Protocol for the generation of human expandable hepatic organoids (EHOs) from iPSCs. (A) Schematic depiction of the differentiation protocol from iPSCs to hepatobiliary organoids. (B) Representative images depicting morphological changes at different stages of the differentiation. From left to right: IPSCs, DE, HB, cystic EHOs and “lobule‐like” EHOs. Scale bars, 200 μM for B1‐3 and 500 μM for B4‐5. (C) Relative gene expression at different stages of the differentiation of endoderm markers, (D) early hepatocyte markers and (E) early biliary specification. Data represents three biological replicates from independent control donors. For C–E, human liver samples were included in the analysis. (F) Immunofluorescent staining of EHOs using albumin (green) on top and HNF4 (green) bottom, with nuclei in blue and cell membrane in red. Scale bar = 100 μm. (G) Upper graph: Urea released in supernatant by organoids (blue) and human precision‐cut liver slices (PCLS) (yellow) in 24 h. Lower graph: Albumin released in supernatant by organoids (blue) in 24 h. Data represents 10 biological replicates for the organoids and 3 biological replicates for the PCLS from independent experiments. All graphs: Error bars indicate SEM.

In order to differentiate iPSCs into hepatic organoids, a previously published protocol [20] was slightly adapted (Figure 1A): to supplement Wnt3a and hRspon1, conditioned medium was used instead of recombinant proteins. At Day 8, the cells were transferred from 2D culture and embedded into BME domes and cultured in expansion medium (EM) for at least 10 days. Morphological changes in the cells were observed at the different stages of the differentiation (Figure 1B). While most of the organoids were cystic and contained clear lumina, more complex structures could also be observed. Some organoids formed “lobule‐like” structures emerging from a central structure (Figure 1B). At this stage, expandable hepatic organoids (EHO) could be cryopreserved and—after thawing—expanded in culture for several passages without any clear morphological changes.

In order to assess cell identity at the different stages of differentiation, the gene expression profile of the organoids was characterized (Figure 1C–E). We defined different developmental stages including iPSCs, definitive endoderm (DE), hepatoblasts (HB), and EHOs (Figure 1A,B), and compared them to human liver biopsies. Endoderm‐specific markers *SOX17*, *FOXA2*, *GATA4*, and *GATA6* were all induced at the DE stage. *FOX2A*, *GATA4*, and *GATA6* stayed higher than in the iPSCs at all stages, while *SOX17* declined in EHOs, resembling the liver reference (Figure 1C). Early hepatic specification markers *TBX3* and *HNF4‐α* were already observed in the DE stage but peaked in the EHOs. For alpha‐fetoprotein (*AFP*), a fetal hepatocyte marker, we observed a peak in expression at the stage of EHOs and barely any presence in the earlier stages, nor in liver tissue. The hepatic marker albumin was not observed until later stages of the differentiation (HB) and showed its maximal expression in the EHOs. Interestingly, the albumin expression was very high in the EHOs and almost comparable to that in human liver (Figure 1D). Finally, the expression of early biliary markers *CK19* and *SOX19* (Figure 1E) illustrates the presence of more than one cell type in the organoids. Together, these results highlight that the differentiation protocol from iPSCs into EHOs recapitulates the early stages of hepatic development.

Different functional assays as well as immunostainings were performed in the EHOs to determine their “liver‐like” state. Immunostainings of the EHOs in culture revealed the expression of more mature hepatic markers *ALB* and *HNF4‐α* (Figure 1F). Finally, the functionality of EHOs was confirmed by the secretion of albumin and urea into the supernatant (Figure 1G).

### 2.3 The Biochemical Phenotype of MCAD Deficiency in EHOs

**FIGURE 2:**
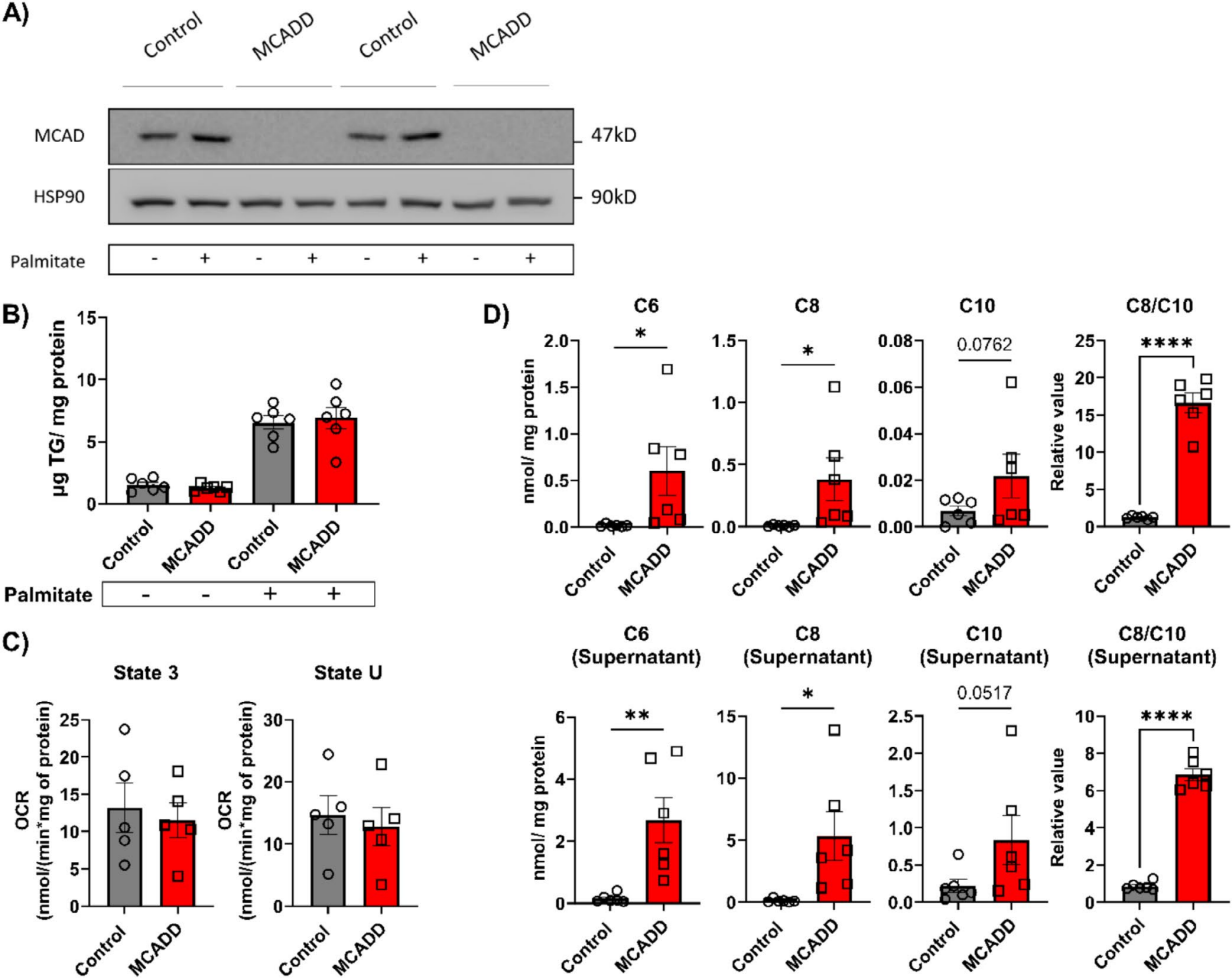
MCADD EHO organoids display an MCADD‐characteristic phenotype in culture. (A) Representative immunoblot images illustrating the lack of MCAD protein in MCADD organoids; data represents 2 MCADD lines and 2 control lines. (B) Intracellular triglyceride levels in control and MCADD organoids. On the left, organoids were stimulated with bovine serum albumin (BSA). The graph on the right depicts the values after treating the organoids with 0.5 mM of BSA‐Palmitate; data represents the mean of 6 biological replicates ± SEM (Unpaired two‐tailed *t*‐test) (C) Oxygen consumption rate in organoids measured at state 3 (stimulated with ADP, octanoyl carnitine and malate) and uncoupled (state U); Data represents the mean of 5 biological replicates ± SEM (Unpaired two‐tailed *t*‐test). (D) Upper panel: Medium chain acyl‐carnitines measured in the organoids incubated with palmitate and L‐carnitine for 24 h (C6, C8, and C10) and C8/C10 ratio; data represents 6 biological replicates from independent cultures ± SEM. Lower panel: Medium‐chain acylcarnitine measured in supernatant (C6, C8, and C10) and C8/C10 ratio. Data represents six biological replicates from independent cultures ± SEM (*p < 0.05, **p < 0.01, ***p < 0.001, ****p < 0.0001, one‐tailed unpaired *t* test).

In order to stimulate fatty‐acid metabolism, we treated the organoids either with bovine serum albumin (BSA) or BSA‐bound palmitate for 24 h. Palmitate is a fatty acid containing 16 carbon atoms and a substrate for mitochondrial fatty‐acid oxidation. Control organoids showed the presence of MCAD protein, both with and without palmitate. In contrast, MCAD was not detectable in organoids from MCADD patients, in either stimulated or unstimulated conditions (Figure 2A), in line with the fact that the c.985A>G (p.K329E) variant destabilizes the protein [34]. Addition of palmitate in the supernatant for 24 h led to a substantial accumulation of triglycerides (TGs) in the organoids. However, no significant difference was observed between control and the MCADD organoids (Figure 2B).

The oxygen consumption rates with octanoyl‐carnitine (C8‐acylcarnitine) as the substrate were in the same range as those reported for primary‐tissue‐derived organoids [35] and the hepatocyte cell line HepG2 [28] (Figure 2C). C8‐acylcarnitine is taken up by cells and imported into mitochondria, where it is converted to octanyl‐CoA (C8‐acyl‐CoA) by carnitine palmitoyltransferase II (CTP2). C8‐acyl‐CoA is a direct substrate of MCAD. Surprisingly, however, the ability of permeabilized organoids to oxidize C8‐acylcarnitine in the presence of ADP and malate did not differ between control and MCADD organoids, nor did uncoupled respiration (Figure 2C). One potential explanation is the overlapping substrate specificity of MCAD with the other dehydrogenases, SCAD (C4‐C6‐acyl‐CoA) and VLCAD (C8‐acyl‐CoA), which may partially compensate for the lack of MCAD [28]. We explored this using a computational model of human mFAO [28], based on detailed enzyme kinetics for all enzymes involved. We simulated the oxygen consumption rate (OCR) of control and MCADD livers when 0–25 μM C8‐acylcarnitine was provided as substrate (Figure S2). In the simulations, a maximum reduction of 30% in OCR was predicted in MCADD compared to the control (at 25 μM). A detailed analysis of the acyl‐CoA dehydrogenation partitioning over the enzymes in the model shows that VLCAD oxidizes C8‐acyl‐CoA, while SCAD takes over C6‐acyl‐CoA oxidation in the absence of MCAD activity (Figure S2). Thus, under the simulated conditions, SCAD and VLCAD would be able to significantly compensate for the lack of MCAD, sustaining the OCR.

Next, we quantified the carnitine profile of the organoids to assess the activation of the mitochondrial fatty‐acid oxidation and analyze the accumulation of medium‐chain acyl‐carnitines, characteristic of MCADD patients. Control and MCADD organoids were incubated with palmitate and L‐carnitine for 24 h and the acyl‐carnitine profile was assessed both intracellularly and in the supernatant. While no changes were observed in short‐ and long‐chain acyl‐carnitines (Figure S3), MCADD organoids accumulated medium‐chain acyl‐carnitines with 6 to 10 carbon atoms (C6, C8, and C10 in Figure 2C,D). Moreover, intracellularly the C8/C10 ratio, an important diagnostic biomarker of MCADD in clinical practice [1], was strongly increased in MCADD organoids (from 11 to 20) (Figure 2D). The same pattern was observed in the supernatant where the C8/10 ratio was upregulated from 6 to 8 (Figure 2D). The values are in the same range as those observed in the plasma of severe MCADD patients (median 13.1), whereas mild MCADD patients (≥ 10% residual MCAD activity) had a median C8/10 ratio of 3.10 and healthy controls 0.8 [1].

### 2.4 Maturation of EHOs Into Mat‐EHOs Increases Peroxisome Abundance

Although peroxisomes are specialized in their ability to oxidize very‐long‐chain, branched, and dicarboxylic fatty acids, they are also capable of oxidizing medium‐chain acyl‐CoA. Given the indications of a higher number of peroxisomes in hepatic cells [35], we decided to first further differentiate the EHOs into mature EHOs (Mat‐EHOs) in order to stimulate peroxisome proliferation.

**FIGURE 3:**
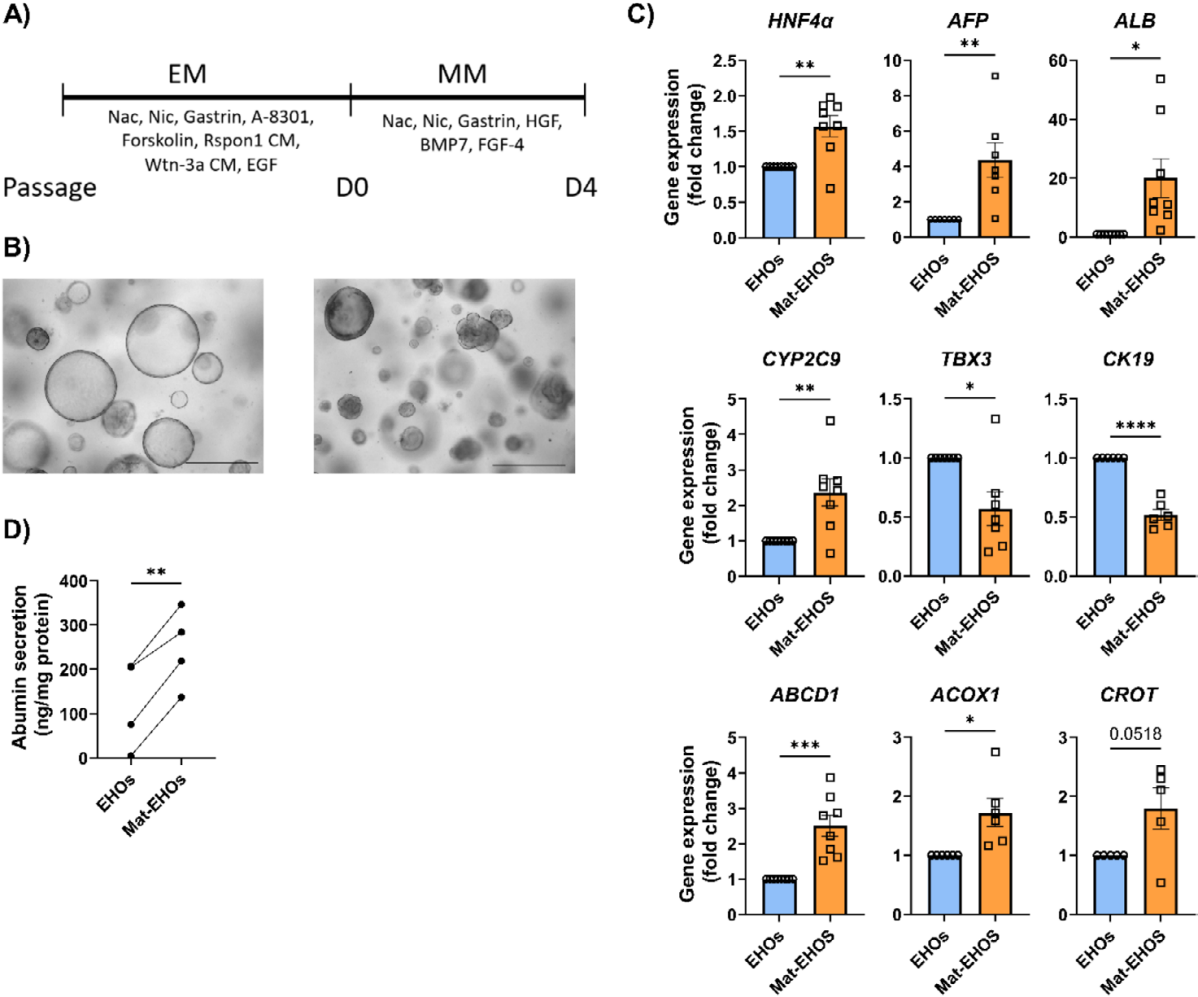
Maturation of EHOs into Mat‐EHOs. (A) Schematic depiction of the maturation protocol (B) Representative brigh;tfield images of EHOs and Mat‐EHOs. Scale bar = 500 μm. (C) Relative gene expression of mature hepatic markers and peroxisomal markers in EHOs and Mat‐EHOs. Data represent eight biological replicates from independent experiments ± SEM (*p < 0.05, **p < 0.01, ***p < 0.001, ****p < 0.0001, two‐tailed unpaired *t* test). (D) Albumin secreted into the supernatant after 24 h in EHOs and Mat‐EHOs. Data represent four biological replicates ± SEM (*p < 0.05, **p < 0.01, two‐tailed unpaired *t* test).

EHOs were kept in expansion medium (EM) for 4–5 days after passage and then transferred to maturation medium (MM) (Figure 3A). Maturation medium contains different cytokines that allow the EHOs to mature into a more hepatocyte‐like state. EHOs in MM underwent morphological changes, shrinking in size and developing thicker outer edges (Figure 3B). Maturation was confirmed by upregulation of mature hepatocyte markers *HNF4‐α*, *AFP*, Albumin, and *CYP2C9*. Matured organoids also showed lower expression of cholangiocyte markers *TBX3* and *CK19*. Moreover, genes coding for enzymes involved in peroxisomal fatty‐acid oxidation (*ABCD1*, *ACOX1*, and *CROT*) were significantly upregulated (Figure 3C). Finally, Mat‐EHOs secreted higher levels of albumin into the supernatant than EHOs, in line with a more hepatocyte‐like phenotype (Figure 3D).

### 2.5 Adaptations in Fatty‐Acid β‐ and ω‐Oxidation in MCADD Mat‐EHO

**FIGURE 4:**
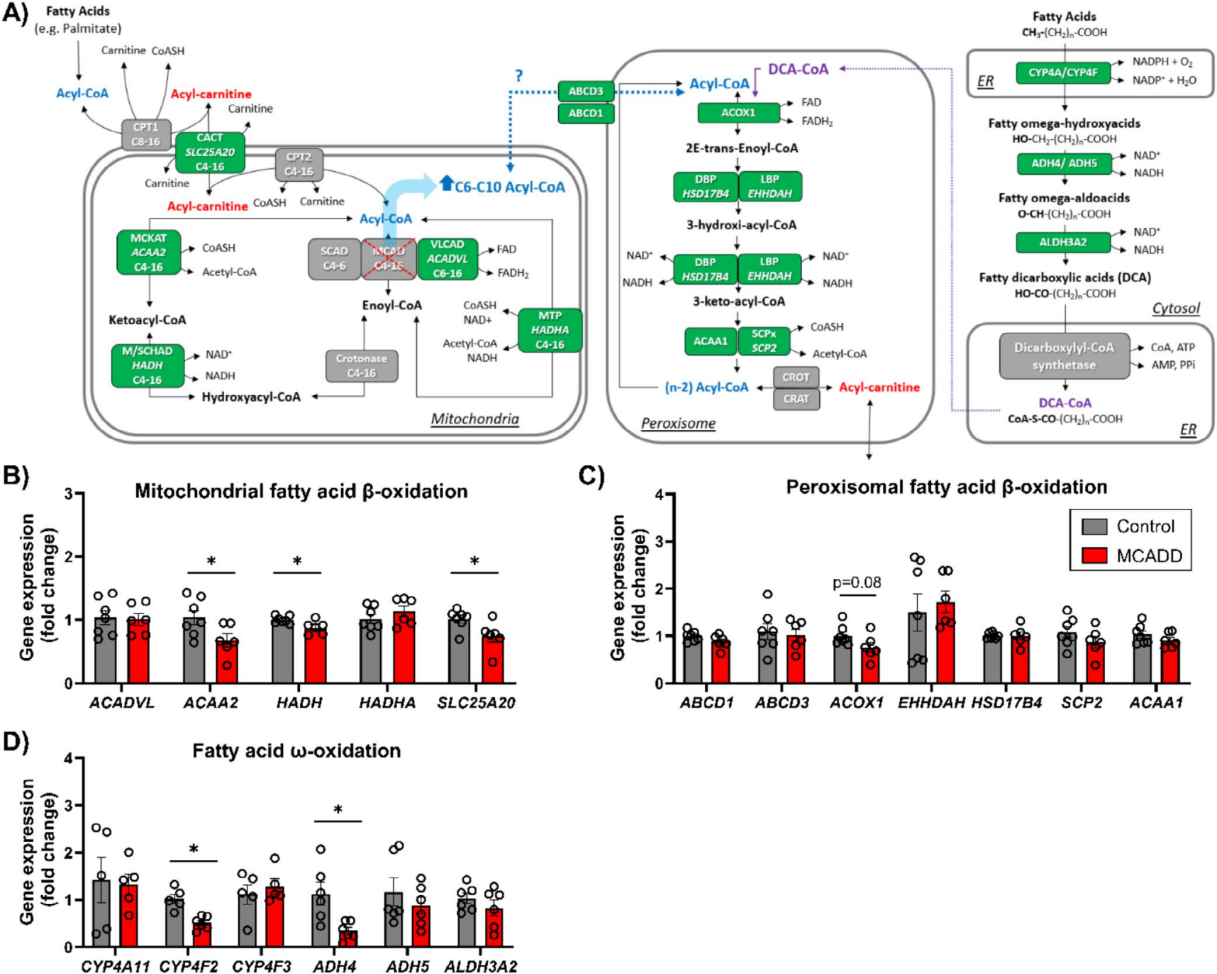
Adaptations of mitochondrial and peroxisomal β‐oxidation and ω‐oxidation in MCADD in Mat‐EHOs. Mat‐EHO organoids were grown in glucose‐free medium supplemented with BSA and L‐carnitine for 24 h. (A) Schematic depiction of the hypothesized interplay between mitochondria and peroxisomes in the β‐oxidation of medium chain fatty acids and fatty dicarboxylic acids (DCA). In green, the targets analyzed in B–D. (B–D) Relative gene expression of genes involved in the (A) mitochondrial β‐oxidation, (C) peroxisomal β‐oxidation, and (D) ω‐oxidation. Data represents six to seven biological replicates for control and five to six for MCADD ± SEM (*p < 0.05, two‐tailed unpaired *t* test). Control (grey) and MCADD (red).

Depending on their chain lengths, fatty acids are β‐oxidized either in the mitochondria or in the peroxisomes [36]. In FAODs excess fatty acids can be channeled into ω‐oxidation, producing fatty dicarboxylic acids (DCAs) as end products, which are subsequently β‐oxidized in the peroxisomes (Figure 4A) [36]. We investigated if fatty acid ω‐oxidation and mitochondrial and peroxisomal β‐oxidation pathways were regulated in response to loss of MCAD protein (Figure 4B–D). Several genes encoding mitochondrial β‐oxidation enzymes were downregulated in the MCADD Mat‐EHOs relative to controls, including medium‐chain ketoacyl‐CoA thiolase (MCKAT) (encoded by *ACAA2*), hydroxyacyl‐coenzyme A dehydrogenase (M/SCHAD) (encoded by *HADH*) and carnitine/acylcarnitine carrier protein (CACT) (encoded by *SLC25A20*) (Figure 4B). Given the ability of peroxisomes to oxidize MCFA, it has been hypothesized that in the absence of MCAD, excess medium‐chain acyl‐CoAs can also be channeled to peroxisomes, where they undergo further β‐oxidation until C6‐acyl‐CoA, which may be shuttled back into the mitochondria to be oxidized by SCAD [29] (Figure 4A). For the peroxisomal β‐oxidation, the genes encoding for the transporters ABCD1 and ABCD3 (ATP binding cassette transporter subfamily D; *ABCD1* and *ABCD3*), the oxidase ACOX1 (acyl‐CoA oxidase 1; *ACOX1*), DBP and LBP (D‐bifunctional protein and L‐bifunctional protein; *HSD17B4* and *EHHDAH*, respectively) and the thiolases ACAA1 and SCPx (*ACAA1* and *SCP2*, respectively) were evaluated (Figure 4C). Apart from a non‐significant decline in *ACOX1* expression, the expression of other genes was not significantly changed in MCADD organoids. Lastly, we assessed ω‐oxidation and found that the genes encoding the enzymes cytochrome P450 family 4 subfamily F member 2 (*CYP4F2*) and alcohol dehydrogenase 4 (*ADH4*), which are involved in the ω‐oxidation of long‐chain fatty acids [37, 38], were downregulated in the MCADD Mat‐EHOs compared to controls (Figure 4D). A similar pattern was observed in the EHOs (Figure S4a).

In conclusion, all the observed adaptations showed downregulation, rather than upregulation of putative compensatory pathways.

**FIGURE 5:**
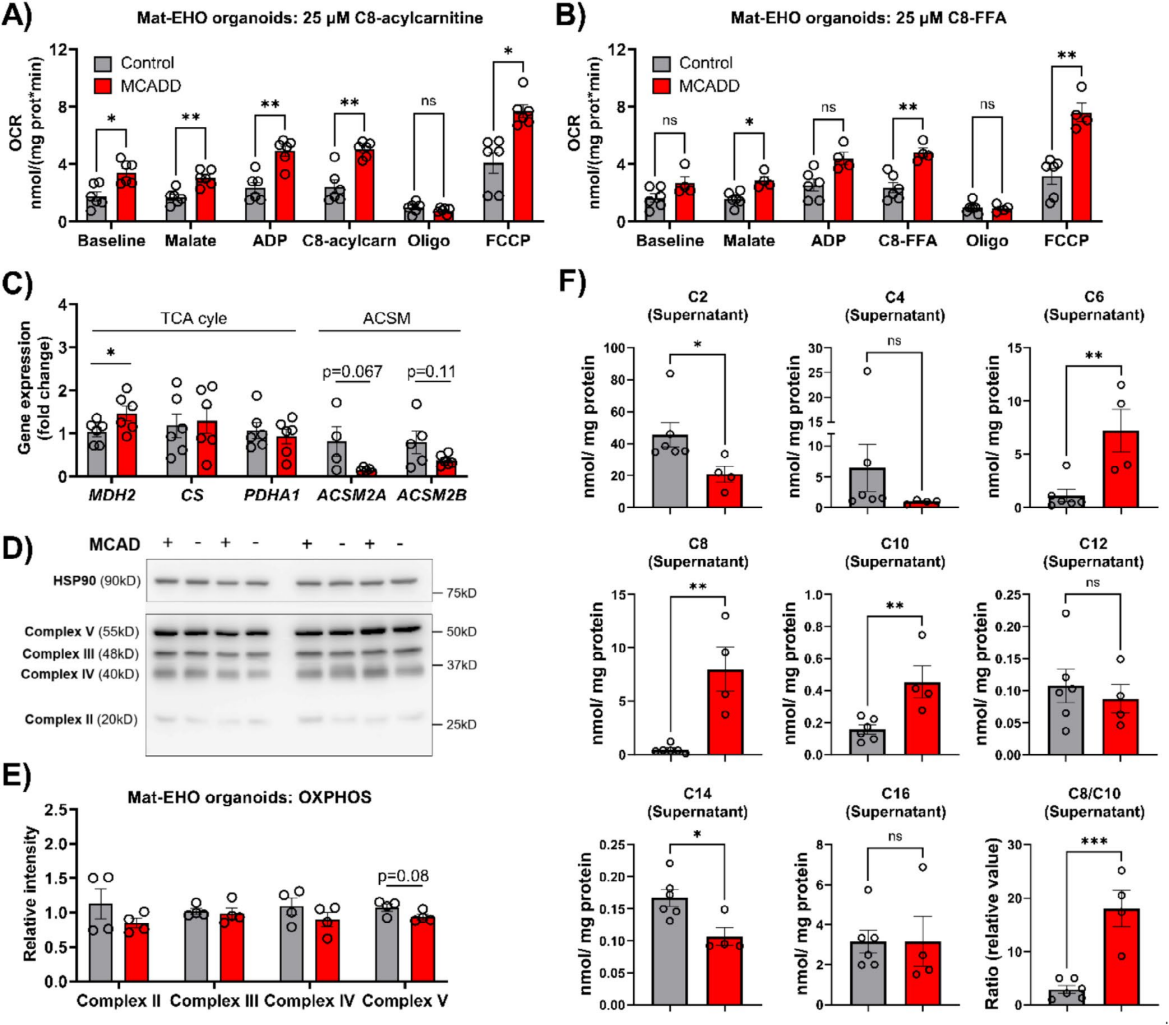
Functional characterization of the mitochondrial β‐oxidation in MCADD and control Mat‐EHOs. Mat‐EHOs were grown in glucose‐free medium supplemented with BSA‐palmitate or L‐carnitine. (A) Oxygen consumption rate (OCR) in organoids measured at baseline, stimulated with malate, ADP and C8‐acylcarnitine (added in steps), and at uncoupled state (FCCP). (B) OCR in organoids measured at baseline, stimulated with malate, ADP and C8‐FFA (added in steps), and at uncoupled state (FCCP). For A, B, data represents the mean of 5–6 biological replicates ± SEM (Two‐way repeated measures ANOVA, followed by Šídák's multiple comparisons test). (C) Relative gene expression of acyl‐CoA synthetase medium‐chain (*ACSM*) and genes involved in the TCA cycle in Mat‐EHOs. The same analysis was performed in EHOs (Figure S4b,c). For *MDH2*, one‐tailed unpaired *t*‐test was applied (*p < 0.05), as we had a specific hypothesis regarding the direction of the effect based on the data obtained in (A, B). (D) Immunoblot images of OXPHOS complexes II, III, IV and V. (E) Quantification of data shown in (D). For C–E, Mat‐EHO organoids were grown in glucose‐free medium supplemented with BSA and L‐carnitine. (F) Acyl‐carnitines measured in supernatant collected after 24 h in glucose‐free medium supplemented with BSA‐palmitate and L‐carnitine. For C–F, data represents four to six biological replicates ± SEM (*p < 0.05, two‐tailed unpaired *t* test); control (grey) and MCADD (red).

Subsequently, we assessed the functionality of the mitochondrial fatty‐acid β‐oxidation in the Mat‐EHOs by high‐resolution respirometry and acylcarnitine profiling (Figure 5). To mimic the fasting conditions, which often trigger symptoms in MCADD patients, we exposed the Mat‐EHO organoids to glucose‐free medium in the presence of BSA‐palmitate and carnitine. A recent study has reported that octanoate (C8‐FFA) is primarily activated to C8‐acyl‐CoA by acyl‐CoA synthetase medium‐chain (ACSM) in the liver, bypassing the carnitine shuttle [39] (Table S3). As C8‐acyl‐CoA is the main substrate of MCAD, we tested both C8‐acylcarnitine and C8‐FFA as substrates in the respirometry assay (Figure 5A,B). The expression of *ACSM* was also assessed in the Mat‐EHOs, showing a downward trend in the MCADD organoids compared to controls, albeit not significant (Figure 5C). Surprisingly, permeabilized MCADD Mat‐EHOs displayed a higher OCR than the controls in almost all conditions (Figure 5A,B). This difference was already observed at baseline and in the presence of malate and ADP, before the addition of fatty acids. Contrary to initial expectations, the OCR of both control and MCADD Mat‐EHOs did not change in response to the addition of either C8‐acylcarnitine or C8‐FFA (Figure 5A,B), which indicated that mFAO was not the main process controlling the OCR. As the tricarboxylic acid (TCA) cycle and oxidative phosphorylation (OXPHOS) can influence the OCR through the generation and O_2_ respiration–coupled oxidation of the electron carriers NADH and FADH_2_, respectively, we investigated whether these processes were regulated in MCADD Mat‐EHOs. First, we assessed the expression of genes encoding the TCA enzymes, citrate synthase (*CS*), malate dehydrogenase (*MDH2*) and pyruvate dehydrogenase (*PDHA1*) (Figure 5C). Consistent with the higher OCR observed in the presence of malate, MCADD Mat‐EHOs also showed upregulation of *MDH2* compared to controls. Subsequently, we assessed the levels of mitochondrial complexes II, III, IV, and V involved in OXPHOS, but no significant differences were detected between MCADD and control Mat‐EHOs (Figure 5D,E). Altogether, our data suggest that the TCA cycle, rather than mFAO, is the main process controlling the OCR in the Mat‐EHOs, and it appears to be upregulated in MCADD compared to controls. Lastly, we quantified the DCA and acylcarnitine profiles of MCADD and control Mat‐EHOs. Unfortunately, the DCA levels were below detection limit. Again, similar to the biochemical phenotype in MCADD patients, medium‐chain acyl‐carnitines accumulated in MCADD and the ratio C8/C10 was strongly elevated (Figure 5F). Interestingly, and unlike in the EHOs, in Mat‐EHOs short‐chain acyl‐carnitine (C2) and long‐chain myristoyl‐carnitine (C14) were decreased (Figure 5F). In agreement with the changes observed in the acyl‐carnitine profile, a recent *in silico* study, based on a detailed kinetic model of human fatty‐acid oxidation, predicted decreased levels of short‐chain and C12‐14 acyl‐CoA and acyl‐carnitines in MCADD [28]. Such decrease has been suggested to be caused by a concomitant depletion in the free coenzyme A (CoASH) levels and, consequently, reduced entry of long‐chain fatty acids into the pathway [28, 40].

### 2.6 CoA Metabolism Does Not Change in MCADD Mat‐EHO

**FIGURE 6:**
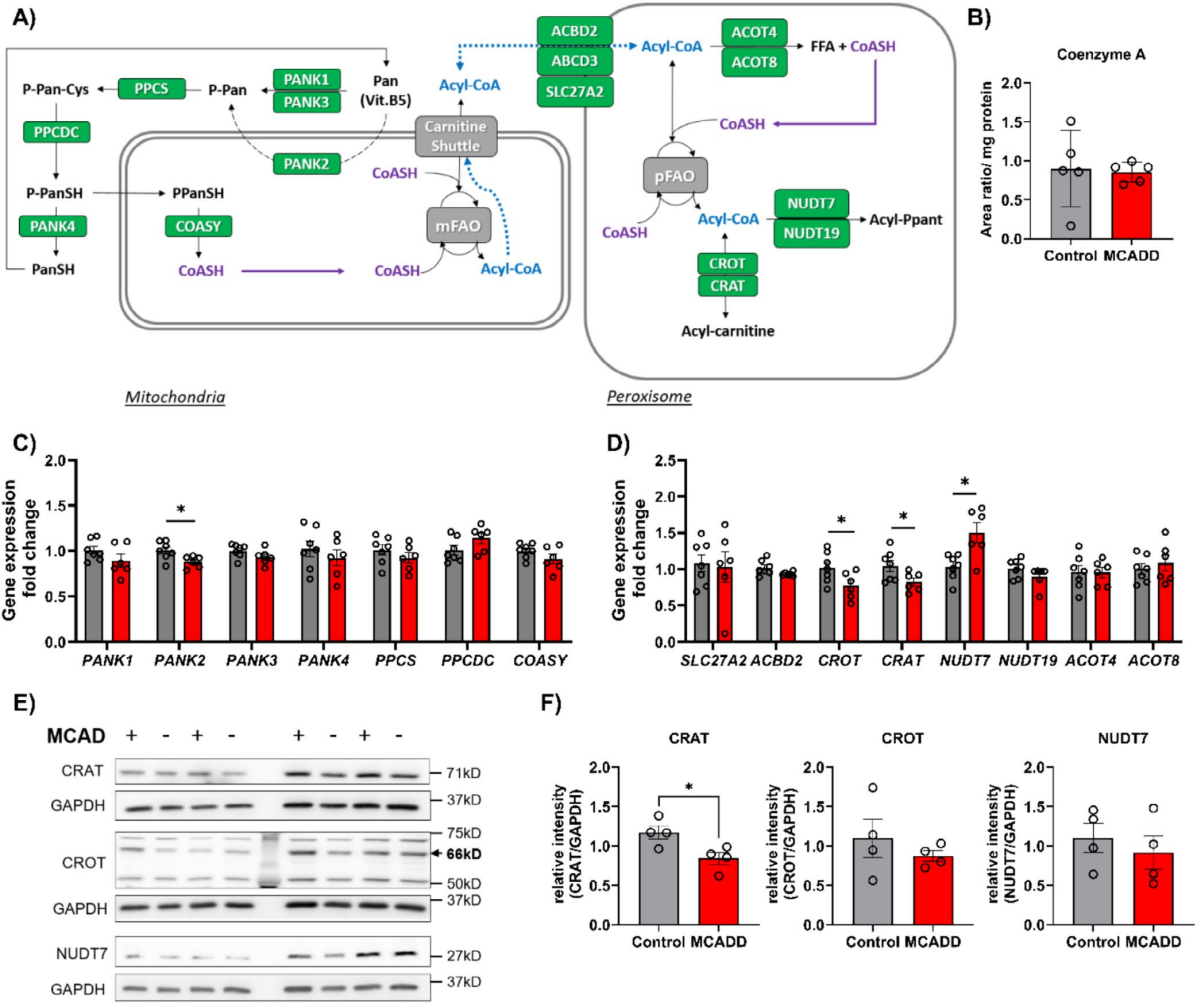
CoA metabolism in Mat‐EHOs. (A) Schematic depiction of the hypothesized effect of MCADD on CoA pools and compensatory mechanism from peroxisomes. In green, the targets analyzed in C–D. (B) Total coenzyme A pools in control (grey) and MCADD (red) organoids. Organoids were grown in glucose‐free medium supplemented with BSA‐palmitate and L‐carnitine. Data represents five biological replicates ± SEM. (C) Relative gene expression of genes involved in CoA biosynthesis. Data represents seven biological replicates for the control group and five for MCADD ± SEM. (*p < 0.05, **p < 0.01, two‐tailed unpaired *t* test). (D) Relative gene expression of genes involved in CoA metabolism. (E) Immunoblot images of CRAT, CROT and NUDT7 in MCADD and control Mat‐EHOs. (F) Quantification of data shown in (E). For C–F, organoids were grown in glucose‐free medium supplemented with BSA and L‐carnitine. Data represents seven biological replicates for the control group and five for MCADD ± SEM (*p < 0.05, **p < 0.01, two‐tailed unpaired *t* test).

CoA is an essential intracellular cofactor involved in several metabolic pathways, and its level is dynamically regulated to adjust to the metabolic state through biosynthesis and turnover [41] (Figure 6A). Furthermore, CoASH as well as acyl‐CoA and acylcarnitine esters have been reported to allosterically regulate the enzymatic activity of pantothenate kinase (PANK), the first enzyme in the CoA biosynthetic pathway [42, 43, 44]. Considering the changes observed in the acylcarnitine profile of MCADD Mat‐EHOs, we interrogated its potential effect on the CoA biosynthesis pathway and total CoA pool.

The total CoA pool (free CoA plus acyl‐CoA) did not differ between MCADD and control Mat‐EHOs (Figure 6B). CoA biosynthesis occurs partly in the cytosol and partly in the mitochondria (Figure 6A). *PANK2*, encoding the mitochondrial PANK paralog, was slightly, but significantly, downregulated in the MCADD organoids (Figure 6C). In addition to CoA biosynthesis, carnitine acyl‐transferases and acyl‐thioesterases (ACOT) also modulate the size of the CoA and acyl‐CoA pools in different intracellular compartments. Peroxisomes also contain several enzymes involved in CoA recycling (Figure 6A), among them two acyl‐thioesterases (ACOT4 and ACOT8), and two carnitine acyl‐transferases: carnitine octanoyl‐transferase (CROT) and carnitine acetyl‐transferase (CRAT), in charge of converting Acyl‐CoAs into their respective acyl‐carnitine form, with the concomitant release of free CoA. MCADD organoids showed substantially lower *CROT* and *CRAT* expression than healthy controls (Figure 6D). *NUDT7*, a gene that encodes a peroxisomal Nudix hydrolase, which hydrolyzes CoA and acyl‐CoA species into 4′pantotheine and acyl‐4′‐pantotheine [45, 46] and plays a role in the metabolism of dicarboxylic acids [32, 47] was clearly upreg in MCADD organoids (Figure 6D, Figure S4d). Finally, we assessed whether the changes in gene expression were reflected in the protein level (Figure 6e,F). Consistent with the mRNA pattern (Figure 6D) and the reduced levels of C2‐acylcarnitine (Figure 5F), CRAT was found to be downregulated in MCADD Mat‐EHOs compared to controls. However, CROT and NUDT7 protein levels showed no differences between the groups.

Taken together, we observe a regulation of some genes involved in CoA metabolism, but an obvious compensatory response could not be identified.

## 3 Discussion

In this paper we have established a patient‐specific iPSC‐derived hepatic organoid system for the study of MCADD. These organoids recapitulated the metabolic profile with high levels of medium‐chain acyl‐carnitines and an elevated C8/C10 ratio that is typical of severe MCADD patients. Furthermore, the Mat‐EHOs expressed pathways that are relevant for future studies of the pathophysiology of the disease. It may not be surprising that we observed only minor adaptations in putative compensatory pathways, since the MCADD organoids were derived from symptomatic patients. Compensatory pathways would rather be expected to be more pronounced in asymptomatic patients. Additionally, catabolic stressors (e.g., glucose starvation, intercurrent infection) in the organoids could have exacerbated the phenotype, given their importance in the manifestation of symptoms in MCADD patients. Nevertheless, we observed minor but notable differences between MCADD organoids and controls in CoA metabolism and the TCA cycle, as evidenced by reduced levels of C14‐acyl‐carnitine, upregulation of *NUDT7* and *MDH2*, and increased OCR in MCADD.

### 3.1 Studying Metabolic Functions in iPSC‐Derived Hepatobiliary MCADD Organoids

Discovery of organoids in 2009 [16] revolutionized the field of biomedical research, providing an alternative to traditional primary hepatocytes. Recent work has focused on the development of organoids from iPSCs. iPSC‐derived hepatobiliary organoids have often been described as close to fetal tissue [25], and advancing their maturity levels to that of liver tissue remains a challenge. Here we compared different maturation stages of the organoids for the study of MCADD. While the original protocol uses a 3‐step procedure that relies on spontaneous aggregation of hepatic single cells [20], we found that this step was hard to reproduce and did not yield enough biomass for metabolic readouts. Therefore, for the purpose of our study, EHOs were matured to Mat‐EHOs (equivalent of “pre‐maturation” stage in the reference paper [20]). At this stage, Mat‐EHOs were found to already exhibit liver‐like phenotypes such as typical hepatocyte mRNA markers, albumin and urea secretion, and the presence of functional mitochondria and peroxisomes.

Despite the functional mitochondrial fatty‐acid β‐oxidation in the generated EHOs and Mat‐EHOs, evidenced by the acylcarnitine profile, these organoids seem to present a great reliance on glucose oxidation. This observation is supported by the respirometry assays, in which neither control nor MCADD Mat‐EHOs responded to the addition of mFAO substrates (C8‐acylcarnitine and C8‐FFA). In other words, the measured OCR was not driven by mFAO but sustained by malate, ADP, and another substrate providing acetyl‐CoA to the TCA cycle. Surprisingly, MCADD Mat‐EHOs even exhibited a higher OCR than controls, which may be explained by an increased TCA cycle flux, consistent with their low dependence on mFAO and the upregulation of *MDH2* in the MCADD organoids. Increased glucose oxidation via glycogenolysis and the TCA cycle, or amino‐acid oxidation, could represent a compensatory mechanism in the MCADD organoids. However, further investigation is needed to test this hypothesis, such as analyzing substrate and protein levels, enzymatic activity, and/or directly assessing metabolic fluxes in the organoids. These observations contrast with previous reports by Lim [48] and Odendaal & Jager [28], who observed a reduced OCR in MCADD fibroblasts (in the presence of glucose) and MCAD‐KO HepG2 cells (in the presence of C16‐acylcarnitine), respectively. Interestingly, in the first study, the reduced OCR was associated with decreased levels of OXPHOS complexes in MCADD fibroblasts [48]. It should be noted that the MCADD fibroblasts described by Lim were obtained from patients carrying different *ACADM* variants than the one described in this study (c.985A>G). Regarding in vivo data, a recent study investigating potential compensatory mechanisms in MCADD found no differences in the mitochondrial OCR with any substrate (pyruvate, C8‐ and C16‐acylcarnitine) nor regulation of the TCA cycle in MCAD‐KO mice treated with low‐ and high‐fat diets [10]. It is unclear, however, if this can be translated to the human situation, since rodents present an additional dehydrogenase, LCAD, which may compensate for the lack of MCAD.

Despite the counterintuitive OCR data, both MCADD EHOs and Mat‐EHOs showed accumulation of medium‐chain acyl‐carnitines (C6‐C10), recapitulating a major biochemical phenotype and confirming active fatty‐acid oxidation. Interestingly, only MCADD Mat‐EHO organoids presented reduced levels of short‐ and long‐chain acyl‐carnitine relative to their control counterparts. These changes are in line with a recent in silico study from our group, based on a detailed computational model of the fatty‐acid oxidation, which predicted reduced levels of short‐ and long‐chain acyl‐CoAs and their corresponding acyl‐carnitines in MCADD [28]. Decreased short‐chain acyl‐CoA or acyl‐carnitine levels are not surprising, since they are downstream of the deficient MCAD enzyme. C14, however, is upstream of MCAD, and at first sight it might therefore be expected to be elevated or unchanged. In the computational model, the reduced C14‐acyl‐CoA could be attributed to a limitation of CoASH availability. CoASH was sequestered into medium‐chain acyl‐CoAs, which strongly accumulated in MCADD. The decrease of free CoA, in turn, limited the entry of new long‐chain fatty acids into the mFAO pathway [28, 40] and thereby led to a reduced C14‐acyl‐CoA. While our data provide experimental support of this non‐intuitive computational prediction, the CoASH availability and its role in the pathophysiology of MCADD warrant further investigation.

Together these data suggest that the maturation step into Mat‐EHO organoids, accompanied by peroxisome and mitochondrial enrichment and a representative acylcarnitine profile, is a relevant step to study the pathophysiological mechanisms of MCADD.

### 3.2 Adaptations of ω‐Oxidation and Peroxisomal β‐Oxidation of Fatty Acids in MCADD

While peroxisomes have been reported to oxidize MCFAs [29], we did not observe any regulation of peroxisomal enzymes involved in the import and oxidation of fatty acids in MCADD organoids, which suggests this pathway was not further activated.

In addition to the oxidation of fatty acids, peroxisomes play a central role in the oxidation of dicarboxylic fatty acids (DCA) [49]. Under high fatty acid (FA) supply, such as fasting and mFAO disorders, excess FAs are channeled to DCA via ω‐oxidation [50, 51]. In MCADD, patients in metabolic crisis have been reported to exhibit accumulation of medium‐chain dicarboxylic fatty acids (MC‐DCA) and high excretion in urine [52, 53]. Excess DCAs can be metabolized by the peroxisomal β‐oxidation [49, 54] or via the activity of nudix hydrolase 7 (NUDT7) [32, 47]. Herewith, the expression of peroxisomal ABCD3 transporter and LBP, both playing major roles in DCA oxidation [55], was not regulated in the MCADD organoids. Zooming into the ω‐oxidation, we confirmed the expression of genes encoding relevant enzymes in the pathway in both EHOs and Mat‐EHOs. Unfortunately, dicarboxylic fatty acids could not be detected in the organoids nor in the supernatant, which may indicate dysfunctional ω‐oxidation or the lack of sensitivity of the chosen method to detect very low concentrations of metabolites.

DCA accumulation in the liver can be toxic, causing inflammation, fibrosis, and death [56]. Recent animal studies suggest that Nudt7 contributes to the regulation of dicarboxylic fatty acid metabolism in the liver [32, 47]. Male mice lacking Nudt7 (Nudt7 −/−) exposed to high‐fat diet showed accumulation of MC‐DCA [32]. The authors proposed a major role of NUDT7 in the regulation of the levels of MC‐DCA. Therefore, we hypothesized that the upregulation in NUDT7 expression in the MCADD organoids may play a role in mitigating and preventing excessive accumulation of MC‐DCA that otherwise could be toxic [56]. However, the change in gene expression was not reflected at the protein level, potentially due to the absence or low levels of DCA in these organoids. Nevertheless, the consistent upregulation of *NUDT7* in both MCADD EHOs and Mat‐EHOs warrants further investigation in vivo.

### 3.3 Conclusion and Outlook

In conclusion, the Mat‐EHO organoids derived from iPSCs of severe MCADD patients show a biochemical phenotype that is characteristic of these patients and provide a good basis for future mechanistic studies into patient‐to‐patient variability. For instance, they can be used to assess the phenotype of genetic variants of the *ACADM* gene that give rise to different residual activities. More interestingly, they can be used to study putative compensatory pathways in asymptomatic patients with a classical c.985A>G and zero residual MCAD activity, for example, in participants of the Fasting Tolerance in MCADD‐infants (FiTtINg MCADD) study (NCT03761693). Furthermore, nutritional interventions and different stressors can be modulated in vitro to study liver‐specific aspects of MCADD pathophysiology and potential therapeutic interventions.

## 4 Methods

### 4.1 Human Fibroblasts

Fibroblasts from patients without documented heritable metabolic diseases (*n* = 2) and also from symptomatic patients with MCADD carrying the classical c.985A>G missense mutation (*n* = 2) were obtained from the Department of Genetics of the University Medical Center Groningen. Patients 5 and 8, previously described in the literature [28], were chosen for this study because of their classical mutation, symptomatology, and normal growth of fibroblasts. Control fibroblasts from C104 and C105 were also described in the literature [28]. All patients were born prior to the implementation of the neonatal MCADD screening in the Netherlands (2007). Both MCADD patients were symptomatic, suffered at least one recorded metabolic crisis, resulting in hospitalization with hypoglycaemia (< 2.6 mmol/L), coma, and/or seizures.

Fibroblasts were cultured in Ham's F‐10 Nutrient Mix (Thermo Fisher Scientific 11550043), supplemented with 10% FCS (Gibco) and 1% penicillin/streptomycin (Gibco).

### 4.2 Generation and Culture of iPSCs From Patient Fibroblasts

Human fibroblasts were reprogrammed into induced pluripotent stem cells following the previously published protocol [33]. iPSCs were cultured in Matrigel (Corning)‐coated plates in mTeSR Plus medium (STEMCELL Technologies). Cells were passaged every 4–6 days, and medium was changed every other day.

### 4.3 Generation of EHOs From iPSCs

iPSCs were differentiated into expandable hepatic organoids (EHOs) following the previously published protocols for hESCs [20, 57] with some adaptations. iPSC cells were cultured in Matrigel‐coated plates and placed in RPMI1640 medium supplemented with 1× B27‐Supplement (Invitrogen), 100 ng/mL Activin A (Peprotech), and 30% Wnt3a‐conditioned medium (kindly provided by Hans Clevers). Wnt3aCM was removed after 24 h, and cells were kept in the same medium supplemented with 100 ng/mL Activin A and 2× B27‐supplement for 48 h. The cells were then transferred to Hepatocyte Culture Medium (HCM) (Lonza, CC‐3198) without EGF and supplemented with 20 ng/mL BMP4 and 10 ng/mL FGF2 (both Peprotech) for the next 5 days. After 5 days, the cells were dissociated using 0.25% Trypsin–EDTA and embedded in BME domes (5000–10 000 cells per 50 μL dome) in Corning 24‐well plates. The cells were cultured in an expansion medium (expansion medium) containing Ad. DMEM/F12 (Gibco) supplemented with 1× Glutamax, 1× HEPES, P/S (all Gibco), 1× B‐27 Supplement, 1× N‐2 Supplement (all Invitrogen), 10 mM Nicotinamide (Sigma Aldrich), 1.25 mM N‐acetylcysteine (Sigma Adlrich), 50 ng/mL EGF (Peprotech), 10uM Forskolin (TOCRIS), 10 nM Iso‐leu gastrin (Sigma Aldrich), 30% Rspon1‐CM (Kindly provided by Calvin J. Kuo) and 10% Wnt‐3a CM.

Medium was changed every 2–3 days. Organoids were passaged every 7–9 days by manually disrupting the BME domes and split in a ratio of 1:8–1:10 depending on the line and supplemented with 10 μM Y 27632 dihydrochloride (Axon Medchem) for 2 days after passaging.

### 4.4 Maturation of EHOs Into Mat‐EHOs

Expandable hepatic organoids were matured into hepatocyte‐like EHOs (Mat‐EHOs) following the previously published protocol [20]. Organoids were kept in expansion medium for 4–6 days after passage and then placed in maturation medium (MM) containing Ad. DMEM/F12 supplemented with 1× Glutamax, 1× HEPES, P/S, 1× B‐27 Supplement, 1× N‐2 Supplement, 10 mM Nicotinamide, 1.25 mM N‐acetylcysteine, 10 nM Iso‐leu gastrin, 50 ng/mL HGF, 25 ng/mL BMP7, and 25 ng/mL FGF4 (all growth factors from Peprotech). Organoids were kept in maturation medium for 4 days prior to collection for downstream analysis.

### 4.5 Organoid Imaging and Immunofluorescence

For brightfield imaging, organoids were imaged using an AxioObserver Z1 compound microscope (Carl Zeiss), 2.5× and 5× objectives, and an AxioCam MRm3 CCD camera (Carl Zeiss).

For immunofluorescence images, organoids were processed following the previously published protocol [58]. After collection with ice‐cold Ad. DMEM/F12, the organoids were kept on ice for 10 min to ensure BME degradation. After centrifugation at 80G at 4°C, the organoids were reconstituted in 4% PFA and kept at 4C for 45 min. After fixation, organoids were permeabilized using PBS containing 0.1% Tween 20 and kept in that buffer for 2 days. Primary and secondary antibody incubations were performed in PBS containing 0.1% Tween 20 and 0.2% BSA. Primary and secondary antibodies are listed in Table S2. Organoids were imaged using an ImageXpress Micro Confocal High‐Content Imaging System (Molecular devices) with a 20× water‐immersion objective (molecular devices). Fifty slices with a 2 μm step size were acquired for each wavelength. Image processing was performed using Fiji v1.8.0.

### 4.6 RNA Isolation, Reverse Transcription and Quantitative Real‐Time qPCR

RNA was isolated from the organoids using RNase easy Kit (Qiagen) as described by the manufacturer. NanoDrop (NanoDrop Technologies) was used to assess the quality and yield of total RNA. M‐MLV Reverse Transcriptase (200 U/μl) (Invitrogen) was used to perform reverse transcription as established by the manufacturer. qPCR was performed in 384‐well format in duplicates (5–10 ng per well) using FastStart Universal SYBR Green Master (Rox) (Sigmal Aldrich) using QuantStudio 7 Flex (Thermo Fischer Scientific). All primer (Integrated DNA technologies Inc) sequences are listed in Table S1. β‐Actin served as endogenous control and was used for normalization.

### 4.7 Hepatocyte Functional Assays

For albumin measurements, the supernatant was collected after 24 h. Levels of albumin in the supernatant were measured with the human albumin ELISA kit (Abcam) as described by the manufacturer using the Synergy H4 Hybrid Microplate Reader (BioTek Instruments Inc).

Urea secretion was assessed in the organoid supernatant using the QuantiChrom Urea kit as described by the manufacturer.

All results were normalized to protein content.

### 4.8 Immunoblotting

The protocol followed for immunoblotting was slightly modified from a previously published paper [59]. Organoids were collected using ice‐cold Ad. DMEM/F12 and kept on ice for 10 min to degrade the BME. Organoids were then centrifuged at *290 g* for 5 min at 4°C and washed with cold PBS prior to another centrifugation step. After the second centrifugation, the organoids were reconstituted in radio immunoprecipitation assay (RIPA) buffer containing 1% IGEPAL CA‐630, 0.1% SDS, and 0.5% sodium deoxycholate in PBS. RIPA buffer was supplemented with Phosphatase Inhibitor Cocktail 2 (Cat. No. P5726) and Cocktail 3 (Cat. No. P0044) and Complete Protease Inhibitor Cocktail (Cat. No. 1186145001) (All Sigma Aldrich). Sonics Vibra cell VCX130 (Sonics & Materials Inc.) was used to sonicate organoid lysates using 4 pulses of 10 s on, 30 s off at an amplitude of 30%. Lysates were then centrifuged at 12 000 rcf for 10 min at 4°C to ensure the precipitation of cell debris. Protein content was determined using Pierce BCA Protein Assay Kit (ThermoScientific) and all samples were adjusted to the lowest concentration value. Lysates were adjusted with Laemmli loading buffer (5×: 60 mM Tris‐Cl pH 6.8, 10% glycerol, 1% SDS, 0.05% Bromophenol Blue, 1% beta‐mercaptoethanol). Protein separation was done in SDS‐PAGE 10%–14% using a Mini PROTEAN Tetra Vertical Electrophoresis Cell system (Bio‐Rad, 1658029FC). For western blot, proteins were transferred to a polyvinylidene difluoride membrane (Immobilon‐P, Millipore).

### 4.9 Fat Isolation and Triglyceride Quantification

Organoids were collected in 1× TBS (137 mM NaCl, 2.7 mM KCl, 66 mM Tris, pH 7.4) in MiliQ water. Fat was extracted in chloroform: methanol in a ratio of 2:1. The levels of hepatic triglycerides were quantitatively determined using the DiaSys Triglyceride FS kit (Holzheim). Results were normalized to protein content.

### 4.10 High Resolution Respirometry

Organoids were collected at Days 8–10 using ice‐cold Ad. DMEM/12 and kept on ice for 10 min. Organoids were then centrifuged at *290 g* at 4°C for 5 min and washed with 2 mL of MiR05 buffer, followed by another spin and finally reconstituted in 600uL of MiR05 buffer, containing 110 mM sucrose, 60 mM potassium lactobionate, 20 mM taurine, 20 mM HEPES, 0.5 mM EGTA, 10 mM KH2PO4, 3 mM MgCl2, and 1 mg/mL bovine serum albumin, at pH 7.1.

Oxygen consumption rate was measured in the organoids using a two‐channel high‐resolution Oroboros Oxygraph‐2k (Oroboros). Organoids were first permeabilized by addition of digitonin (0.02 mg/mL). The maximal coupled respiration was measured in the presence of 1 mM ADP, 25 μM octanoyl‐carnitine, and 2 mM malate (state 3). Basal respiration was determined by the subsequent addition of 0.002 mg/mL of oligomycin to block ATP synthase (state 4). Finally, uncoupled respiration (state U) was measured after subsequent administration of 1.5 μM carbonyl cyanide p‐(trifluoromethoxy) phenylhydrazone (FCCP). Oxygen consumption rates were normalized to protein concentration.

### 4.11 Acylcarnitines Measurements

EHO organoids were incubated with 0.5 mM BSA‐conjugated palmitate (Sigma‐Aldrich, P9767) and 2 mM l‐carnitine (Sigma Aldrich) for 24 h prior to collection in PBS. Acylcarnitines were measured both in supernatant and intracellularly following the published protocol [60].

### 4.12 Total CoA Measurements Using HILIC‐MS/MS Analysis

Mat‐EHO organoids were incubated in glucose‐free maturation medium supplemented with 0.5 mM BSA‐conjugated palmitate and 2 mM l‐carnitine for 24 h. Organoids were collected in ice‐cold medium and washed 2× in ice‐cold PBS. Samples were prepared as described elsewhere [61]. Briefly, the pellet was reconstituted in 600 μL MilliQ H_2_O, and the lysate was sonicated using a Sonics Vibra cell VCX130 (25 s, 50% amplitude, two times). Lysates were centrifuged at 14000 rpm for 15 min at 4°C. In a new tube, 80 μL Tris (2‐carboxyethyl)phosphine hydrochloride (10 mM) was added to 400 μL supernatant and incubated for 15 min at room temperature. Next, samples were spun down (14 000 rpm, 15 min, 4°C). In a new tube, 40 μL ammonia solution was added to 400 μL supernatant (1.25% v/v) and incubated shaking at 500 rpm at 60°C for 60 min. Lastly, samples were dried using a SpeedVac (Eppendorf) and reconstituted in 100 μL ice‐cold 80% methanol.

Coenzyme A was extracted by a two‐step protocol using chloroform/methanol/water based on the Bligh and Dyer approach [62]. The detailed sample preparation and HILIC‐MS/MS protocol are described in detail in the literature [63].

### 4.13 Human Precision‐Cut Liver Slices (hPCLS)

PCLS with approximately 250–300 μM thickness were prepared using a Krumdieck Tissue Slicer (Alabama Research and Development), as described elsewhere. Individual slices were kept in culture for 24 h in Williams E medium (WE), containing 25 mM glucose, 0.5% BSA, 1 mM l‐carnitine, and gentamycin (Invitrogen). The plates were kept under a continuous supply of 80% O_2_/5% CO_2_, shaking at 70 rpm.

Human liver tissue was collected from a transplantation donor at the University Medical Center Groningen (UMCG, Netherlands). The liver was used for research purposes after being rejected for transplantation with the approval of the Research Ethics Committee of UMCG.

### 4.14 Statistical Analyses

All results are expressed as mean ± standard error of the mean (SEM). Analyses were performed using GraphPad Prism Software Version 9.02 (Graphpad Software). Statistical significance was determined as *p value < 0.5, **p value < 0.01, and ***p value < 0.001; no indication means no significant changes (ns). For iPSC work, biological replicates are considered individual donors. For organoid work, due to the low number of patients, biological replicates are considered organoids coming from the two same patients or healthy controls that were at least one passage apart.

This work was supported by the European Union's Horizon 2020 research and innovation programme under the Marie Skłodowska‐Curie Actions Grant Agreement PerICo No 812968 and PoLiMeR No 812616; the Stichting De Cock‐Hadders and the Stichting Beatrix Kinderziekenhuis.

## Disclosure

Animal Rights: This article does not contain any studies with animal subjects performed by the any of the authors.

## Consent

The use of stored patient fibroblasts was approved by the Medical Ethical Committee of the University Medical Center Groningen and confirmed according to the Dutch law.

All procedures followed were in accordance with the ethical standards of the responsible committee on human experimentation (institutional and national) and with the Helsinki Declaration of 1975, as revised in 2000 (5). Informed consent was obtained from all patients for being included in the study.

Additional informed consent was obtained from all patients for which identifying information is included in this article.

## Conflicts of Interest

The authors declare no conflicts of interest.

## Supporting information


Data S1.
