# Supplementary material for: iPSC‐Derived Liver Organoids as a Tool to Study Medium Chain Acyl‐CoA Dehydrogenase Deficiency
Source: J Inherit Metab Dis. 2025 Apr 8;48(3):e70028. doi: 10.1002/jimd.70028 (PMC11978564; doi:10.1002/jimd.70028)
Supplement: Supplementary file 1 — Data S1. [file JIMD-48-0-s001.docx]

**
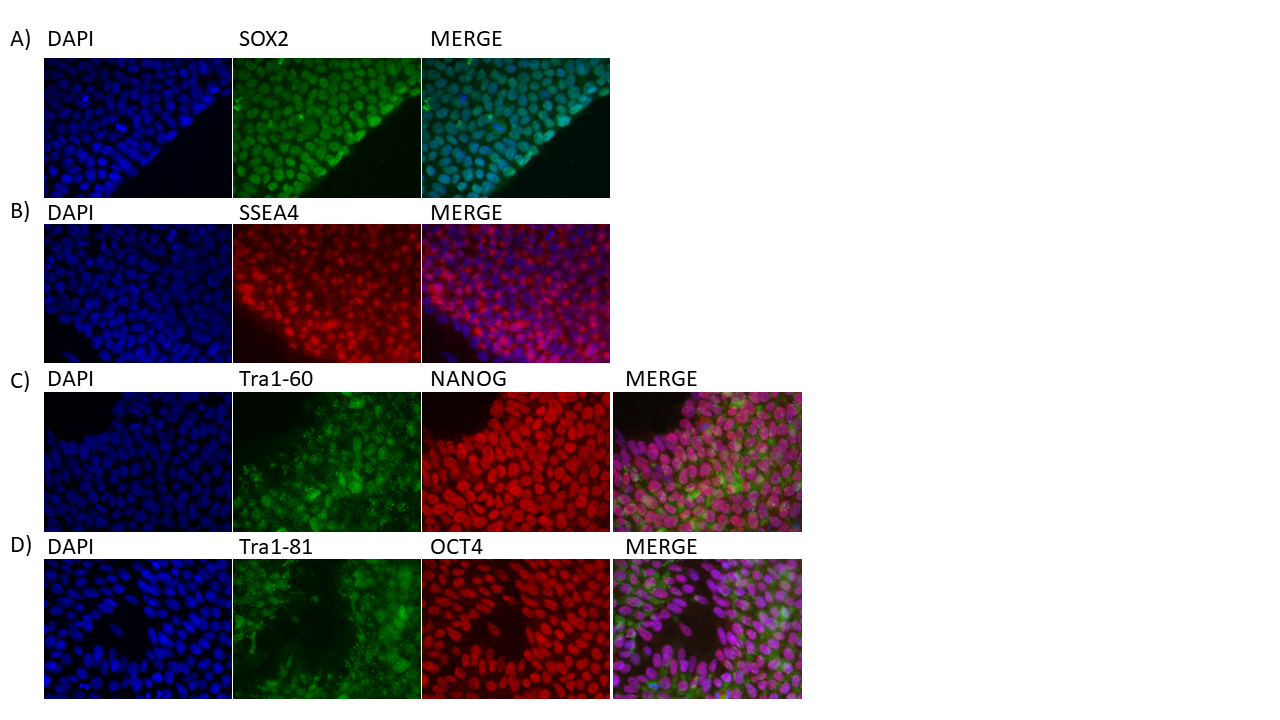
**

**Supplementary Figure S1. Expression of iPSC markers in iPSC control line.** Immunofluorescent staining of DAPI (blue) and (**A**) SOX2 (green), (**B**), SSEA4 (red), (**C**) Tra1-60 (green) and NANOG (red) and (**D**) Tra1-81 (green) and OCT4 (red).

**
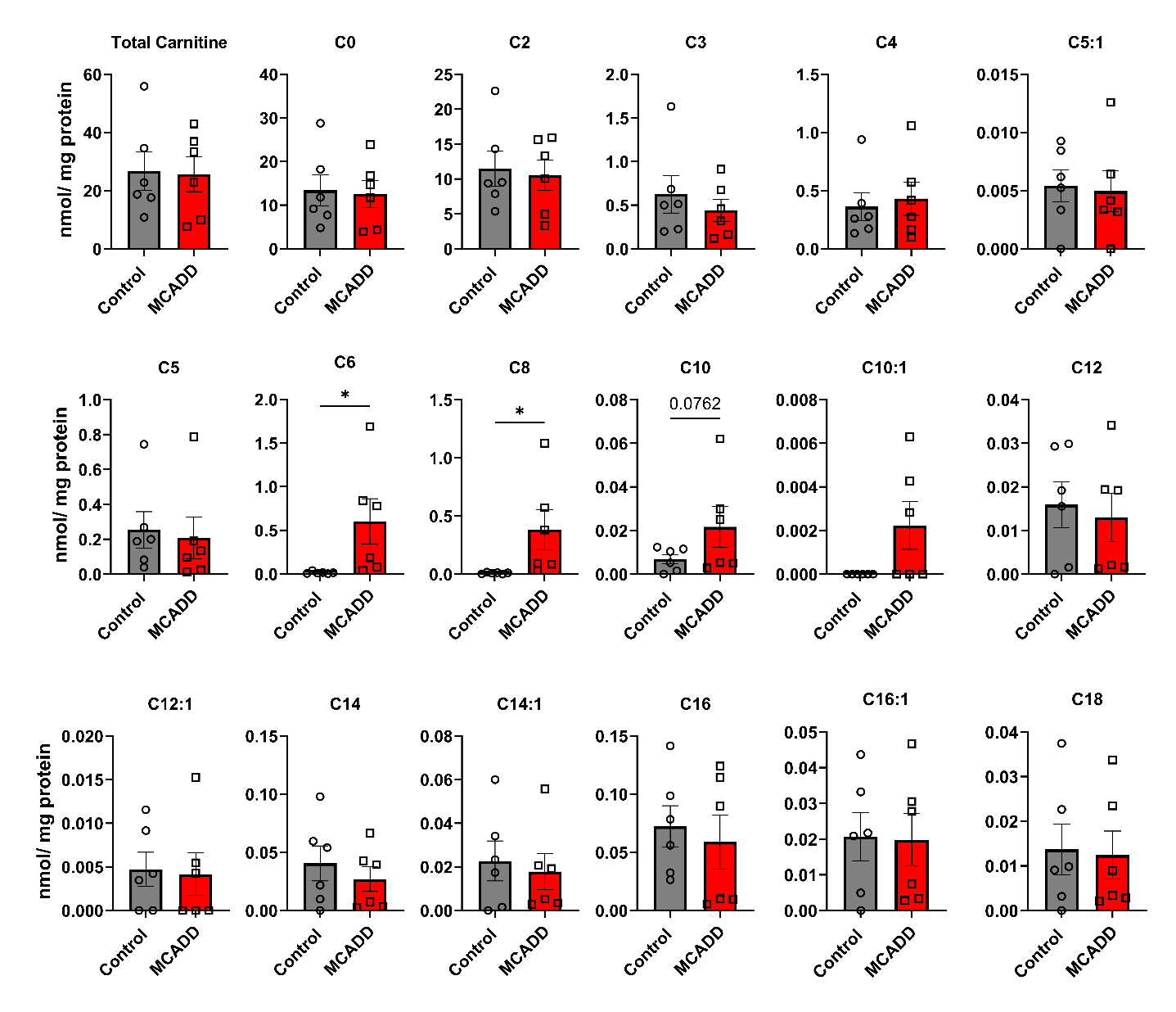
**

**Supplementary Figure S2. Intracellular acyl-carnitine profile of control (grey) and MCADD (red) EHOs.** Data represents 6 biological replicates ± SEM. (*P<0.05 one-tailed unpaired t-test).

**
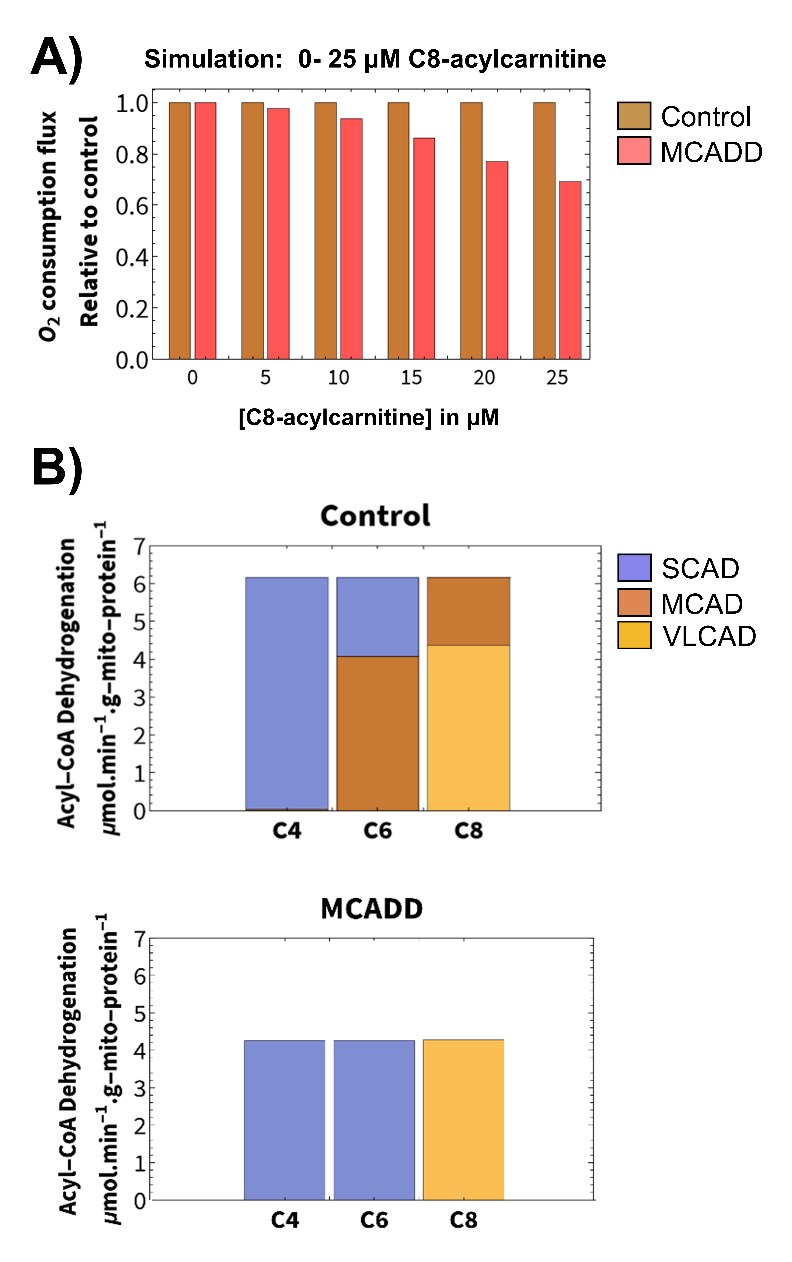
**

**Supplementary Figure S3. Computational simulations of mFAO in control and MCADD human liver.** (**A**) Simulation of oxygen consumption flux in control (brown) and MCADD (pink) liver using 0- 25 μM C18-acylcarnitine as starting substrate. (**B**) Simulation of the acyl-CoA dehydrogenation partitioning among the SCAD, MCAD and VLCAD enzymes in control and MCADD using 25 μM C18-acylcarnitine as starting substrate. The simulations were done using the kinetic model of mFAO published by Odendaal and Jager^1^.

**
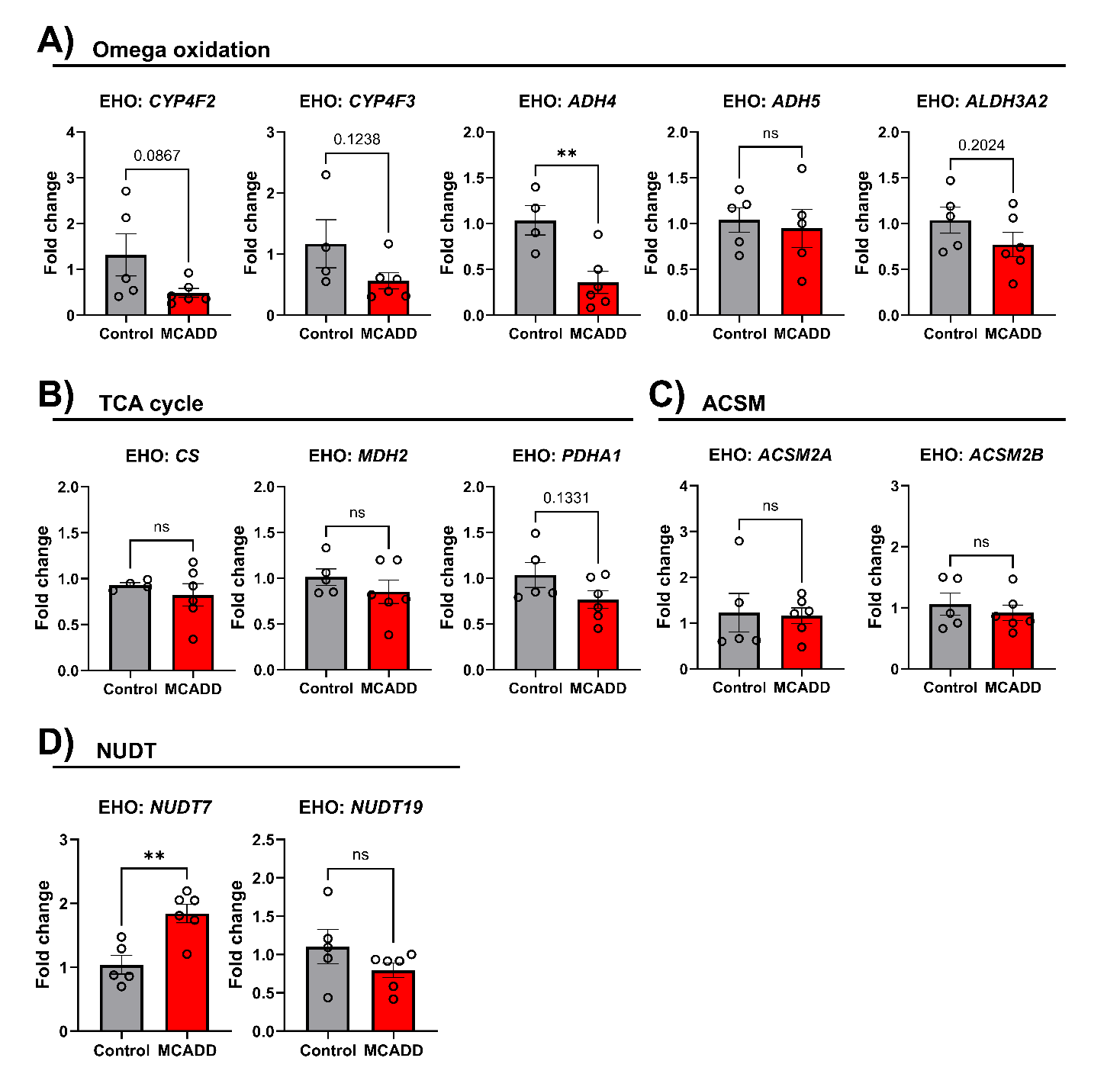
**

**Supplementary Figure S4. Gene expression analysis of ω-oxidation, TCA cycle and ACSM in control and MCADD EHOs.** EHO organoids were grown in glucose-free medium supplemented with BSA and L-carnitine for 24 hours. **(A-D)** Relative gene expression of genes involved in **(A)** ω-oxidation, **(B)** the TCA cycle, **(C)** ACSM, and **(E)** NUDT. β-Actin was used as a housekeeping gene in the data normalization and analysis. Data represents 4-6 biological replicates ± SEM. (*P<0.05, two-tailed unpaired t test). Control (grey) and MCADD (red).

**Supplementary Table 1. List of primer sequences used in RT-qPCR**

| **Gene Name** | **Forward and reverse primer sequence (5’- 3’)** |
| --- | --- |
| *SOX17* | Fwd: CGCACGGAATTTGAACAGTA  Rev: GGATCAGGGACCTGTCACAC |
| *FOXA2* | Fwd: ACTACCCCGGCTACGGTTC  Rev: AGGCCCGTTTTGTTCGTGA |
| *GATA4* | Fwd: CCTCTTTCTCAGCAGAGCTGTA  Rev: CTCTGCTACAGCCAGTAGGATT |
| *GATA6* | Fwd: CCCACAACACAACCTACAGC  Rev: GCGAGACTGACGCCTATGTA |
| *TBX3* | Fwd: TTTCACAATTCTCGGTGGATGGTGGC  Rev: ACGACTTTGGACATGCACTGTTCC |
| *HNF4α* | Fwd: ACTACATCAACGACCGCCAGT  Rev: ATCTGCTCGATCATCTGCCAG |
| *Albumin* | Fwd: GAGACCAGAGGTTGATGTGATG  Rev: AGTTCCGGGGCATAAAAGTAAG |
| *AFP* | Fwd: CTTTGGGCTGCTCGCTATGA  Rev: GCATGTTGATTTAACAAGCTGCT |
| *CK19* | Fwd: ACGACCATCCAGGACCTGCGG  Rev: TCCCACTTGGCCCCTCAGCGTA |
| *SOX9* | Fwd: ACTTGCACAACGCCGAG  Rev: CTGGTACTTGTAATCCGGGTG |
| *ABCD1* | Fwd: CCTTCTGGAACGCCTGTGGTAT  Rev: TTCCAAGGCTGCCTTCTTCACG |
| *ABCD3* | Fwd: GTTCCTTTAGCAACGCCAAATGG  Rev: CTCTTTCCGCAGCCATTTGGAC |
| *NUDT7* | Fwd: CTCCGTCCTTTTGCCATTGGTG  Rev: TGTCTGTAGGGTCACGCTTACC |
| *NUDT19* | Fwd: GCACCACTCGCCGCTTTGACA  Rev: GTTGCCTCTGATGGAGATGACC |
| *SLC27A2* | Fwd: GTGGAGAAAGATGAACCTGTCCG  Rev: CTGAGCCTTTGCTCCAGCATAG |
| *ACBD2* | Fwd: GCTGCCAGCAAGGATGACTCAA  Rev: GCTTTCTCCTCTACTCCACCAG |
| *ACOT4* | Fwd: CTTTGCCACGTTGGCTCTAGCT  Rev: CCTAGAGAAATGCCCAAAAGCCC |
| *ACOT8* | Fwd: GCTGACCACTGGATGCTCTATG  Rev: AGGTCACAGCTAGGACTCCATC |
| *ACOX1* | Fwd: GGCGCATACATGAAGGAGACCT  Rev: AGGTGAAAGCCTTCAGTCCAGC |
| *EHHADH* | Fwd: CGGAGCATCGTGGAAAACAGCA  Rev: CCGAGTCTACAGCAATCACAGG |
| *SCP2* | Fwd: GACAAGGTGCAACGCTGGTTGA  Rev: CCAGCAGAGTTCTGCACACTGA |
| *ACAA1* | Fwd: GACAGGTCATCACGCTGCTCAA  Rev: CCAGGGTATTCAAAGACGGCAG |
| *HSD17B4* | Fwd: GAGAATGCCAGCAAGCCTCAGA  Rev: GCTGTAGACGTTGCACGACTAG |
| *ACADS* | Fwd: CACGCCTTTCACCAGTGGTGAC  Rev: CACGCCTTTCACCAGTGGTGAC |
| *ACADVL* | Fwd: TAGGAGAGGCAGGCAAACAGCT  Rev: CACAGTGGCAAACTGCTCCAGA |
| *ACAA2* | Fwd: GGCACTGAAGAAAGCAGGACTG  Rev: GTGACCCAAAGCAATGGCTCCT |
| *HADH* | Fwd: TCCGTTGTCCACAGCACAGACT  Rev: GGAGGAAGTGTTGCTGGCAAAG |
| *HADHA* | Fwd: GCCGACATGGTGATTGAAGCTG  Rev: GGAGAGCAGATGTGTTACTGGC |
| *SLC25A20* | Fwd: ACCGAGTTTGCCTGGACAACCT  Rev: CCCAAAGAAGCACACGGCAAAC |
| *CROT* | Fwd: CTAGTGAGGAGCGAACTCGATG  Rev: CCTCTGGTGTTACATGTGGACTG |
| *CRAT* | Fwd: CCTACAGACCAACAAGGAGCCT  Rev: TGCATCTAGGCACACGGTGAAG |
| *CYP2C9* | Fwd: GACATGAACAACCCTCAGGACTTT  Rev: TGCTTGTCGTCTCTGTCCCA |
| *PANK1* | Fwd: AGGTGTCAGCATTCTAGCCGTG  Rev: GGTCTCACAACCAGTCAGCAAG |
| *PANK2* | Fwd: CGTGGAGATAGCACCAAAGTGG  Rev: CAGGTCCTCTTTACTGACAGCC |
| *PANK3* | Fwd: TTGCCAGGTTGGGCTGTAGCAT  Rev: GCACACATTCGTGCCACAGAAC |
| *PANK4* | Fwd: TCGTGGATTCCTACAGCGAGTG  Rev: CTGTCCCTCTAAGGAGTAGCTC |
| *PPCS* | Fwd: TCCTGGCAGTAGAGTTCACCAC  Rev: GGCATTTCAGAGACAGGAACATAG |
| *PPCDC* | Fwd: CAAGAAGCTGGTGTGCGGAGAT  Rev: GTCAACTCTGCTGGAAGCCACT |
| *COASY* | Fwd: TGAGGTGTGGACTGCTGTCATC  Rev: TGGCTCTGTTCCACAAGCTGCT |
| *CYP4A11* | Fwd: CATGGCAGACTCTGTACGAGTG  Rev: CTGATGGCTGAAGGCACACTTC |
| *CYP4F2* | Fwd: GACAGCCATTGTCAGGAGAAACC  Rev: TGCAGGAGGATCTCATGGTGTC |
| *CYP4F3* | Fwd: CCACCTACATCAAGCCTGTGCT  Rev: GGCTCCACTTTTCACCAGCACT |
| *ADH4* | Fwd: CCTTGACTGTGCAGGTGGATCT  Rev: GTCAATCCTTTGCTACCAGCAGC |
| *ADH5* | Fwd: GTAAACCCATCCAGGAAGTGCTC  Rev: TGTGACATGCCTCAAGTGCTGC |
| *ALDH3A2* | Fwd: ACTGATAGGAGCCATCGCTGCA  Rev: GCTCCGTGGTTTCCTCAACACC |
| *CS* | Fwd: CACAGGGTATCAGCCGAACCAA  Rev: CCAATACCGCTGCCTTCTCTGT |
| *MDH2* | Fwd: CTGGACATCGTCAGAGCCAACA  Rev: GGATGATGGTCTTCCCAGCATG |
| *PDHA1* | Fwd: GGATGGTGAACAGCAATCTTGCC  Rev: TCGCTGGAGTAGATGTGGTAGC |

Fwd, forward primer; Rev, reverse primer.

**Supplementary Table 2. List of primary and secondary antibodies used in immunoblotting.**

| **Name** | **Company** | **Catalogue number** | **Dilution** |
| --- | --- | --- | --- |
| ACOX1 | Abcam | Ab184032 | WB 1:1000 |
| B-actin | Sigma-Aldrich | A5441 | WB 1:1000 |
| PMP70 | Sigma-Aldrich | P0497 | WB 1:1000 |
| Albumin | R&D systems | MAB1455 | IF 1:100 |
| MCAD | Abcam | AB92461 | IF 1:100 |
| CK19 | Cell Signaling | 4558 | IF 1:200 |
| Epcam | Invitrogen | 17-5791-82 | IF 1:200 |
| HNF4-α | Cell Signaling | 3113 | IF 1:1000 |
| Hoechst 33342 | Life Technologies | H3570 | 2.5μM |
| CROT | Protein Tech | 16801814 | WB 1:1000 |
| CRAT | Atlas Antibodies | HPA022815 | WB 1:1000 |
| NUDT7 | Thermo-Fisher Scientific | PA5-70718 | WB 1:1000 |
| Total OXPHOS | Abcam | ab110413 | WB 1:1000 |
| HSP90 | Cell Signaling | 4874S | WB 1:1000 |
| Alexa Fluor 555 Phalloidin | Cell Signaling | 8953S | IF 1:50 |
| Alexa Fluor 488 goat anti-mouse IgG (H+L) | Invitrogen | A11029 | IF 1:300 |
| Alexa Fluor 488 goat anti-rabbit IgG (H+L) | Invitrogen | A11034 | IF 1:300 |

**Supplementary Table 3. Substrate-specific V_max_ values of human ACSM1.** The V_max_ values of ACSM1 can be compared to the V_max_ of the enzymes involved in the mFAO; V_max,CPT2_ is 0.28 µmol/min/mg-mito-protein; V_max,VLCAD_ is 0.076 µmol/min/mg-mito-protein; V_max,MCAD_ is 0.038 µmol/min/mg-mito-protein ^2^.

| **V_max_**  **nmol/min/mg-purified protein**  **(“unknown purity”)** | **V_max_**  **µmol/min/mg-purified protein**  **(“unknown purity”)** | **V_max_**  **µmol/min/mg-mito-protein*** | **Substrate** |
| --- | --- | --- | --- |
| 9 | 0.009 | 0.00009 | benzoate |
| 24 | 0.024 | 0.00024 | butyrate |
| 8 | 0.008 | 0.00008 | decanoate |
| 118 | 0.118 | 0.00118 | hexanoate |
| 0.15 | 0.00015 | 0.0000015 | laurate |
| 2 | 0.002 | 0.00002 | phenylacetate |

*Assuming ACSM1 is 1% of mitochondrial protein mass.

**Supplementary References**

1. Odendaal, C. *et al.* Personalised modelling of clinical heterogeneity between medium-chain acyl-CoA dehydrogenase patients. *BMC Biol* **21**, (2023).

2. Vessey, D. A., Kelley, M. & Warren, R. S. Characterization of the CoA ligases of human liver mitochondria catalyzing the activation of short-and medium-chain fatty acids and xenobiotic carboxylic acids. *Biochimica et Biophysica Acta (BBA) - General Subjects* (1999).
